# DKA and new-onset type 1 diabetes in Brazilian children and adolescents during the COVID-19 pandemic

**DOI:** 10.20945/2359-3997000000433

**Published:** 2022-01-14

**Authors:** Thais Milioni Luciano, Mariana Peduti Halah, Mariana Teresa Alves Sarti, Vitor Gonçalves Floriano, Benedito Antônio Lopes da Fonseca, Raphael Del Roio Liberatore, Sonir Rauber Antonini

**Affiliations:** 1 Universidade de São Paulo Faculdade de Medicina de Ribeirão Preto Departamento de Pediatria Ribeirão Preto SP Brasil Divisão de Endocrinologia Pediátrica, Departamento de Pediatria, Faculdade de Medicina de Ribeirão Preto, Universidade de São Paulo, Ribeirão Preto, SP, Brasil; 2 Universidade de São Paulo Faculdade de Medicina de Ribeirão Preto Departamento de Clínica Médica Brasil Divisão de Doenças Infecciosas, Departamento de Clínica Médica, Faculdade de Medicina de Ribeirão Preto, Universidade de São Paulo, Brasil

**Keywords:** DKA, Type 1 diabetes, Sars-CoV-19, coronavirus pandemic

## Abstract

We assess the severity and frequency of diabetic ketoacidosis (DKA) in new-onset type 1 diabetes mellitus (T1D) patients and in patients with previous diagnosis of T1D in a referral Brazilian university hospital in the first five months of the COVID-19 pandemic. We also compare the data with data from pre-pandemic periods. Forty-three new-onset T1D patients were diagnosed between April and August of the years 2017, 2018, 2019, and 2020. During the COVID-19 pandemic, the number of new-onset T1D was over twice the number of new-onset T1D in the same period in the three previous years. All the 43 patients survived and are now on outpatient follow-up. We also compared the characteristics of the T1D patients hospitalized between April and August of the years 2017, 2018, and 2019 (32 hospitalizations) to the characteristics of the T1D patients hospitalized between April and August/2020 (35 hospitalizations; 1 patient was hospitalized twice in this period). Fourteen of the 34 patients admitted during the pandemic presented with COVID-19-related symptoms (any respiratory symptom, fever, nausea, vomiting, and diarrhea), but only one had positive SARS-CoV-2 RT-PCR test. Samples from 32 out of these 34 patients were assayed for SARS-CoV-2 antibodies, and four patients were positive for total antibodies (IgM and IgG). In agreement with recent reports from European countries, we observed increased frequency of DKA and severe DKA in new-onset and previously diagnosed T1D children and adolescents in a large referral public hospital in Brazil in the first five months of the COVID-19 pandemic. The reasons for this outcome might have been fear of SARS-CoV-2 infection in emergency settings, the more limited availability of primary healthcare, and the lack of school personnel’s attention toward children’s general well-being.

## INTRODUCTION

The 2020 coronavirus pandemic is an unprecedented health emergency in modern times. While the demand for medical care by patients with acute respiratory syndrome caused by SARS-CoV-2 has increased in emergency settings, the search for medical care for other causes has declined steeply (1). Reports mainly from developed countries have speculated that the fear of COVID-19 contamination may have delayed the search for medical assistance, thereby aggravating the clinical conditions of patients with other diseases, such as type 1 diabetes mellitus (T1D) (2). Herein we assess the severity and frequency of diabetic ketoacidosis (DKA) in new-onset type 1 diabetes mellitus (T1D) patients and in patients with previous diagnosis of T1D in a referral Brazilian university hospital in the first five months of the COVID-19 pandemic and compare these data with data from pre-pandemic periods.

## MATERIALS AND METHODS

This descriptive study collected data from the medical records of T1D patients assisted at the Pediatric Endocrinology Service of the Ribeirao Preto Medical School University Hospital (University of São Paulo-Brazil). This hospital complex is part of the Brazilian publicly funded unified healthcare system known as SUS, and it is the tertiary referral center for a population of approximately 3.5 million in a regional geographical area in the state of Sao Paulo, Southeastern Brazil. In this region, around 70% of the population uses the public health system, whereas 30% is covered by private health insurance. This hospital is also the regional emergency and tertiary reference hospital for pediatric endocrinology.

Data from patients diagnosed with T1D between April and August of the years 2017, 2019, and 2019 (pre-pandemic period) and between April and August/2020 (COVID-19 pandemic period) were obtained and analyzed. The number of new cases of T1D per 1,000 patients seen in the pediatric emergency department was calculated. The same months of 2017-2020 were analyzed to compare the incidence of T1D in the same service in different years and to compare our data with the results of studies that used a similar period in other countries.

We also compared information from patients with previous diagnosis of T1D that required hospitalization between October/2019 and February/2020 (pre-pandemic period) and between April and August/2020 (pandemic period). These months were was chosen because data were available from the institution.

The following data were investigated: number of new-onset T1D, number of decompensated patients with previous diagnosis of T1D, frequency of DKA in these patients, severity and time to recover from DKA, frequency of hypokalemia, age, sex, time elapsed since T1D diagnosis, time elapsed since the last follow-up visit (for the patients with previously diagnosed T1D), and weight z-score. Comparisons were made between the pre-pandemic and pandemic periods. Symptomatic patients were submitted to RT-PCR for SARS-CoV-2, and the presence of SARS-CoV-2 antibodies was prospectively evaluated.

New-onset T1D was defined by glycemia greater than or equal to 7 mmol/L (126 mg/dL) in fasting plasma or greater than 11.1 mmol/L (200 mg/dL) in a random measurement, associated with polyuria, polydipsia, polyphagia, and weight loss in previously healthy individuals aged less than 18 years, with or without DKA in the first presentation, and in need of using insulin to control hyperglycemia. Patients who were diagnosed before the age of 12 months, who had family history of diabetes before the age of 35 years over three generations, who had associated conditions suggesting the use of any medication, or who had a disease or syndrome associated with diabetes were not included. When clinical diagnosis was unclear, diabetes-associated autoantibodies (GAD and IA2) were collected. New-onset was considered when the diagnosis of T1D, with or without DKA, was made between April and August of the years 2017, 2018, 2019, and 2020.

DKA was diagnosed in patient with glycemic equal or greater than 13.8 mmol/L (250 mg/dL) and serum pH lower than 7.3 or serum bicarbonate lower than 15 mmol/L. Severe DKA was defined as serum pH lower than 7.1 or serum bicarbonate lower than 5 mEq/L. Hypocalemia was defined by serum potassium levels lower than 3.5 mEq/L. All the patients included in this analysis were first attended in the pediatric emergency department to confirm or to exclude DKA, and then they were transferred to the pediatric endocrinology ward.

The means, percentages, and variability were calculated, and the chi-squared test and Fisher’s exact test were applied accordingly.

This study was registered in the Plataforma Brazil and the Ethics Committee of the Ribeirao Preto Medical School University Hospital approved this study under the CAAE protocol number 37130820.5.0000.5440.

## RESULTS

Forty-three new-onset T1D patients were diagnosed between April and August of the years 2017, 2018, 2019, and 2020 (Table 1). In the first five months of the COVID-19 pandemic, the number of new-onset T1D was over twice the number of new-onset T1D in the same period of the three previous years. All the 43 patients survived and are now on outpatient follow-up.

**Table 1:** Characteristics of new-onset T1D patients from April to August of 2017, 2018, 2019, and 2020

Clinical Features		Year			
		2017 (n=8)	2018 (n=9)	2019 (n=8)	2020 (n=18)
New onset T1D cases/1,000 patients seen in pediatric emergency department		5.2	5.4	4.1	9.9
Age at diagnosis (years)		8.9	7.9	7.4	7.6
Median (range)		(4.9 – 13)	(1.1 – 13.3)	(2.1 – 14.5)	(1.2 – 15.5)
Age (years)					
	< 6	1	3	4	7
	≥ 6	7	6	4	11
Female (n)		4	5	2	9
Weight Z-score		0.1	-0.1	0.6	0.2
Median (range)		(-2.3 – 2)	(-2.4 – 1.6)	(-1.4 – 3.2)	(-4.6 – 2.9)
DKA at diagnosis (n)		1	6	2	12
Severe DKA^a^		0	3	0	6
Frequency of hypokalemia^b^		3	5	1	11

DKA: diabetic ketoacidosis.

a Severe DKA: serum pH lower than 7.1 or serum bicarbonate lower than 5 mEq/L.

b Hypokalemia: plasma potassium levels lower than 3.5 mEq/L.

We also compared the characteristics of the T1D patients hospitalized between April and August of the years 2017, 2018, and 2019 (pre-pandemic period, 32 hospitalizations) to the characteristics of the T1D patients hospitalized between April and August/2020 (pandemic period, 35 hospitalizations; 1 patient was hospitalized twice in this period) (Table 2).

**Table 2:** Characteristics of T1D inpatients in the pre-pandemic and pandemic periods

Clinical Features		Period		p-value
		N		
		Pre-pandemic (n=32)	Pandemic (n=35)	
Age at diagnosis (years)		11.9	10.4	*
Median (range)		(1.6 – 17)	(1.25 – 18.6)	
Age (years)				
	< 6	2	7	p=0.09
	≥ 6	30	28	
Female (n)		21	21	p=0.63
Weight Z-score		-0.2	-0.01	*
Median (range)		(-5.1 – 2.2)	(-4.6 – 2.9)	
Hospitalization				
New-onset T1D (n)		6	18	p=0.01
Previous diagnoses		26	17	**
	Time since T1D diagnosis (years). Median (range)	5.7 (2–13.7)	3.1 (2.7 – 16)	
	Time since last follow-up visit (months). Median (range)	1.5 (0.2 – 12)	3 (0.2 – 7.5)	
DKA				
New-onset T1D with DKA (n)		5	12	p=0.07
New-onset T1D with severe DKA (n)		2	9	p=0.03
Total of DKA in hospitalizations (n)		11	22	p=0.01
Total severe DKA^a^ in hospitalizations (n)		4	12	p=0.03
Hours for DKA resolution (hours)		10.8	10.9	*
Median (range)		(4 – 20)	(2 – 34)	
Electrolytic disturbance				
Frequency of hypokalemia^b^		9	16	p=0.46

T1D: type 1 diabetes; DKA: diabetic ketoacidosis.

a Severe DKA: serum pH lower than 7.1 or serum bicarbonate lower than 5 mEq/L.

b Hypokalemia: plasma potassium levels lower than 3.5 mEq/L.

* The test is not convenient in this case.

** During the pre-pandemic period, some patients were called for elective hospitalization, that’s why the test was not applied on this occasion.

Data were compared by Fisher’s exact test in the items “Age range”, “New-onset T1D with severe DKA”, and “Total severe DKA in hospitalizations” and by the chi-squared test in the other items.

Fourteen out of the 34 patients admitted during the pandemic presented with COVID-19-related symptoms (any respiratory symptom, fever, nausea, vomiting, and diarrhea), but only one had positive SARS-CoV-2 RTPCR test. Samples from 32 out of these 34 patients were assayed for SARS-CoV-2 antibodies, and four patients were positive for total antibodies (IgM and IgG). The patient with positive SARS-CoV-2 RT-PCR test was one of the four patients who tested positive for total antibodies but had no different hospitalization profile. Three of the latter four patients had previous diagnosis of T1D; only one was diagnosed with new-onset T1D. The new-onset T1D patient presented with DKA, but it was not severe. As for the three patients with previous diagnosis of T1D, one had DKA, one had severe DKA, and one had decompensation without DKA, but their hospitalization profiles were not different compared to the other patients.

## DISCUSSION

In the first five months of the COVID-19 pandemic, we observed higher frequency and severity of DKA in new-onset T1D children and adolescents, as well as a higher number of T1D diagnosis in a large referral hospital in Southeastern Brazil. Similar findings were reported in Germany and London-UK (3,4). A multicentric Italian report also noted increased severity and frequency of DKA, but the number of new-onset T1D did not increase when compared to 2019 (5).

To date, there is no known relationship between the SARS-CoV-2 infection and the development of T1D or DKA. However, it has been speculated that the number of new cases has increased during the pandemic. An association between SARS-CoV-2 and DKA, especially severe DKA, is not evident, either. Here, only one out of 14 patients submitted to RT-PCR tested positive for SARS-CoV-2. In addition, SARS-CoV-2 total antibodies were positive in this and three other patients (12%). Thus, in most of our patients, DKA and T1D cannot be attributed to COVID-19.

We believe that the increased frequency of DKA, especially severe DKA, during the pandemic period is related to delayed search for medical care due to fear of being contaminated with SARS-CoV-2 in emergency settings. The lower availability of primary healthcare might have contributed to this outcome. School closures may have been another reason for this finding given that teachers and school personnel were prevented from paying attention to students’ well-being.

In agreement with recent reports from European countries, we also observed increased frequency of DKA and severe DKA in new-onset and previously diagnosed T1D children and adolescents in a large referral public hospital in Brazil. Additionally, the number of T1D cases apparently increased, but this finding has yet to be confirmed by longer and more detailed observation.

## References

[1] Scaramuzza A, Tagliaferri F, Bonetti L, Soliani M, Morotti F, Bellone S (2020). Changing admission patterns in paediatric emergency departments during the COVID-19 pandemic. Arch Dis Child.

[2] Baum A, Schwartz MD (2020). Admissions to Veterans Affairs Hospitals for Emergency Conditions During the COVID-19 Pandemic. JAMA.

[3] Kamrath C, Mönkemöller K, Biester T, Rohrer TR, Warncke K, Hammersen J (2020). Ketoacidosis in Children and Adolescents With Newly Diagnosed Type 1 Diabetes During the COVID-19 Pandemic in Germany. JAMA.

[4] Unsworth R, Wallace S, Oliver NS, Yeung S, Kshirsagar A, Naidu H (2020). New-Onset Type 1 Diabetes in Children During COVID-19: Multicenter Regional Findings in the U.K. Diabetes Care.

[5] Rabbone I, Schiaffini R, Cherubini V, Maffeis C, Scaramuzza A (2020). Diabetes Study Group of the Italian Society for Pediatric Endocrinology and Diabetes. Has COVID-19 Delayed the Diagnosis and Worsened the Presentation of Type 1 Diabetes in Children?. Diabetes Care.

